# Psychiatric Health Laws in Pakistan: From Lunacy to Mental Health

**DOI:** 10.1371/journal.pmed.0020317

**Published:** 2005-09-20

**Authors:** Ahmed Ijaz Gilani, Umer Ijaz Gilani, Pashtoon Murtaza Kasi, Murad Musa Khan

## Abstract

Recent changes in mental health services and in the laws governing treatment if mentally ill individuals are encouraging, but further improvement is needed.

The South Asian country of Pakistan is the sixth most populous nation in the world. Although modern-day Pakistan came into being only 58 years ago, it is heir to a rich historical heritage spanning thousands of years, first records of which date back to the 4,000-year-old Indus valley civilization. The sociodemographic and socioeconomic characteristics of Pakistan today are shown in Table 1; the major features are a high fertility rate, a correspondingly large, young, and predominantly rural population, and a poor economy.

**Table 1:**
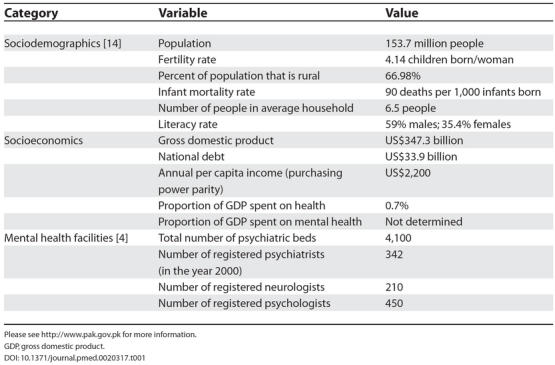
Socioeconomic and Demographic Variables and Psychiatric Facilities in Pakistan

Please see http://www.pak.gov.pk for more information. GDP, gross domestic product.

One of the major health-care problems of the country is mental illness. A systematic review of risk factors, prevalence, and treatment of anxiety and depressive disorders in Pakistan found that the overall mean prevalence of these disorders in the community was 34% (range is 29%–66% for women and 10%–33% for men) [1]. Factors positively associated with these disorders were female sex, middle age, low level of education, financial difficulty, being a housewife, and relationship problems—suggesting that social factors play an important part in the aetiology of anxiety and depression in Pakistan. Other major mental health problems are developmental disorders, psychosis, and drug abuse, although credible estimates for these are lacking.

Islam plays a major role in determining the value system of Pakistani society. On the one hand, society is generally contemptuous of and biased against individuals who are mentally ill [2,3]. On the other hand, good treatment of individuals who are mentally ill is deemed greatly desirable under the society's strong religious and ethical values.

In this article, we examine the infrastructure of mental health services in Pakistan and the Pakistani laws that govern treatment of individuals who are mentally ill. We look critically at how these laws have changed over time. Changes in these laws reflect deeper changes in Pakistani society's attitude towards individuals who are mentally disordered. We stress the need for further improvement in these laws and suggest that Pakistani mental health laws should meet international standards for the treatment of mentally ill people.

## Mental Health Infrastructure

There are many players and factors involved in the access, provision, delivery, functioning, and uptake of mental health services in Pakistan (Figure 1; Table 1). Awareness about mental illness is still poor in Pakistan. Such illness is generally attributed to supernatural causes—it is considered to be a curse, a spell, or a test from God.

**Figure 1:**
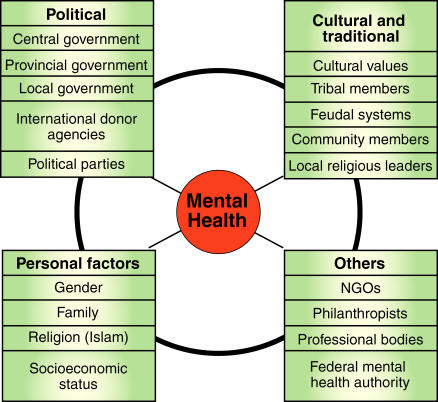
The Players and Factors Involved in the Access, Provision, Delivery, Functioning, and Uptake of Mental Health Services in Pakistan (Image: Aslam Bashir, Aga Khan University)

Those who experience mental illness often turn first to religious healers, rather than mental health professionals, since patients and their families tend to have great faith in these healers. Religious healers use verses from the Koran to treat patients. Next, patients turn to traditional and alternative healers, who are also popular in Pakistani society.

Help from the mainstream health-care system is usually sought late in the course of the illness. In Pakistan's health-care system, a pyramidal model is followed, starting with primary health care at the bottom (Figure 2). However, the referral system is inefficient and, particularly in the case of individuals who are mentally ill, patients are usually taken by their families directly to tertiary or specialist hospitals, rather than to primary-care practitioners. It is, however, important to note that many mental illnesses can be treated and managed by primary-care practitioners. The private sector also plays a major role in providing psychiatric care. For those who can afford it, private psychiatric care is an option frequently used.

**Figure 2:**
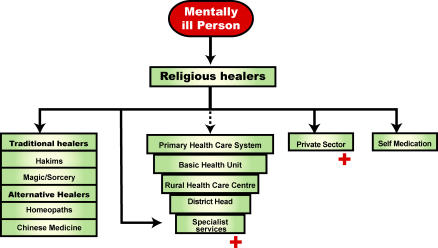
The Usual Model of Seeking Help for Mental Health Illnesses in Pakistan (Image: Aslam Bashir, Aga Khan University)

## Recent Improvements in Provision

Pakistan has come a long way since it gained its independence in 1947, when there were only three psychiatric hospitals in the country. Today around 20 medical colleges support psychiatric wards. At the moment, there are some 4,100 beds in the public and private sector and about 342 practicing psychiatrists, mostly located in major cities (Table 1) [4]. Behavioural sciences and psychiatric training form an essential part of undergraduate medical training.

The National Mental Health Programme, developed in 1986, aims at achieving universal provision of mental health and substance-abuse services by incorporating them into primary health care. Via this programme, primary-care physicians are being trained, and training manuals are being developed for lady health visitors (a type of health worker who provides a variety of services to urban and rural communities, including basic nursing care, maternal and child health services, and training of community workers [5]). In addition, junior psychiatrists are being trained in community mental health. The importance of including spiritual healers in the mainstream health-care and referral system has also been recognised by the National Mental Health Programme, as they are frequently the ones having first contact with individuals who are mentally ill.

## Pakistani Law and Mental Illness

The law has important implications for the lives of all citizens, including those who are mentally ill. The laws governing the treatment of mentally ill people give a clear indication of a country's attitude towards such people. The relationship between a society's attitude and the law is a dynamic one, and a two-way affair.

In Pakistan, until 2001, the major source of laws relating to individuals who are mentally ill was the Lunacy Act of 1912 [6] (Box 1), enacted by the colonial government, at the time, for the whole of British India. After the partition, Pakistani law continued to be based on the relics of its colonial past, although sporadic changes were brought about in the light of drastically changed conditions and the requirements of an Islamic republic. The Lunacy Act of 1912, however, like most other laws, remained in effect, despite occasional protests by the medical profession and society at large.

**Box 1. The Lunacy Act of 1912**

The Lunacy Act [6] was enacted in 1912 for British India. Until recently, it was the most important piece of psychiatric legislation in Pakistan. The statute is divided into four major parts dealing with definitions of crucial terms, rules pertaining to reception, care, and treatment of individuals who are mentally ill, and procedural rules for establishing whether or not an individual is mentally ill. Even a cursory glance at the statute reveals it as woefully inadequate and obsolete for the needs of a modern state. In 2001, the act was replaced by the Pakistan Mental Health Ordinance [7].

On February 20, 2001, the Pakistan Mental Health Ordinance came into effect [7]. The Lunacy Act of 1912 consequently stood repealed. The 2001 ordinance has brought about significant changes in the law “relating to mentally disordered persons with respect to their care and treatment and management of their property and other related matters” [7], as the preamble of the ordinance boldly proclaims.

The ubiquitous presence of ordinances is a peculiar feature of Pakistani law. Ordinances are presidential orders, tantamount to valid law, passed in emergency situations or in the absence of a sitting Parliament. Theoretically, they lapse after three months if opposed by an act of Parliament. In reality, ordinances form an important part of Pakistani law, and like the Pakistan Mental Health Ordinance, permanently hold as much legal value as any act passed by Parliament.

## How Did the 2001 Ordinance Change the Law?

Before 2001, one of the most striking features of the Pakistani law regarding mentally disordered people was the sustained usage of archaic, imprecise, and often undefined terms. The term “lunacy” is a classic example because, despite being shunned by the psychiatric profession for being inhumane and imprecise, it still formed a part of the outdated Lunacy Act's title and was used, along with its derivatives, throughout the act. Lunatic was defined as “an idiot or person of unsound mind” (section 4), a definition that, on account of its vagueness, has also given rise to much case law [8,9].

The 2001 law has discarded such outmoded and imprecise terms as “lunatic”, “criminal lunatic” (an oxymoron, given that “lunatics” cannot be held responsible for their acts), and “asylum”, and has provided its own more comprehensive set of definitions. The ordinance uses the term “mental health” as a part of its title and defines the converse—mental disorder—as “mental illness, including mental impairment, severe personality disorder, severe mental impairment, and any other disorder or disabling of mind…” (section 2(1)(m) of [7]). For all of these categories of mental disorder, comprehensive definitions have also been provided. For example, severe personality disorder is described in the 2001 law as persistent disorder or disability of mind (whether or not it includes significant impairment of intelligence), which results in abnormally aggressive or seriously irresponsible conduct on the part of the person concerned.

The term “mentally disordered prisoner” replaces “criminal lunatic”; similarly, instead of “asylum”, “health facility” and “psychiatric facility” are used wherever suitable. A very pragmatic step forward has been the introduction of a definition of “informed consent” for treatment. In light of the new definition, consent would only be considered valid when it is informed, that is, when the patient (or guardian or nearest relative, in case of a minor) has been adequately informed of the purpose, nature, likely effects, and risks of the treatment, including the likelihood of its success, any alternative, and the costs to be incurred. By addressing this pertinent issue, an important gap in the law has been bridged.

Terminology deeply reflects the mood of those who use it. The adoption, by Pakistani society and law, of more scientifically appropriate, precise, and humane terms indicates that a more empathetic attitude has replaced an earlier attitude of summary dismissal and a lack of understanding towards individuals who are mentally disordered. This is, indeed, a positive development.

An important development brought about by the new law is the establishment of the Federal Mental Health Authority, comprising seven “eminent psychiatrists of at least 10 years standing” (section 3(3)(v) of [7]) and seven other members, largely bureaucrats. This body has been given the responsibility of overseeing the state of mental health provision in the country, setting up national standards of care and treatment, and performing a host of other tasks.

But instead of making immediate changes needed by the mental health system of the country, this authority makes only promises. Its objectives are long term and to an extent vaguely defined. The effect that this institution will have on the lives of individuals who are mentally disordered is hard to judge from what the law says about the institution; the effect depends, rather, on how the institution performs in the future.

## Fewer Days of Forced Detention

Under the Lunacy Act of 1912, the combined effect of sections 13–16 was to allow the detention of people alleged to be “lunatics” for a period of ten days, extendable by the magistrate's permission to a maximum of 30 days, before an actual inquiry was held to establish the detainee's mental status. Given the rampant corruption and widespread abuse of power endemic in the Pakistani legal system, this provision was bound to be exploited. The recent trend in legal reform in Pakistan has been towards restricting police powers of arrest and detention. This trend reflects the realisation that the police have a reputation for abusing their powers.

In the new 2001 ordinance, section 19(2) clearly limits the period of forced detention under the above-mentioned circumstances to a maximum of 72 hours. During this time period, examination by a psychiatrist or the psychiatrist's nominated medical officer has to be ensured, and necessary arrangements must be made for starting care and treatment. It is hoped that this will prevent the widespread abuses of the law that often occurred before 2001.

A patient who is mentally ill on leave from a psychiatric facility may be ordered to be brought back by the magistrate in the area on the advice of the treating psychiatrist.

## Criminal and Civil Liability

One of the most important legal issues that arise with regard to people who are mentally disordered is the issue of the extent of criminal and civil liability. In British law, for instance, individuals who are mentally disordered qualify for the “defence of insanity and automatism”, which leads to a legally stipulated reduction in their liability for criminal offences (see Book 4, chapter 2 in [10]). Similarly, in Pakistani civil law, individuals who are mentally disordered do not have the capacity to enter into valid contracts and, hence, cannot be held liable for breach of contract (section 3(3)(v) of [7]).

The new ordinance, just like its precursor, leaves the crucial question of the extent of criminal and civil liability of people who are mentally ill unanswered, which means that the law on this matter has to be derived from other sources of criminal and civil law. The new ordinance, therefore, has had no bearing whatsoever on one of the most crucial questions of law pertinent to individuals who are mentally disordered: how will it be determined that someone is or is not mentally disordered?

## Human Rights

The latter half of the 20th century has seen the rise of the doctrine of human rights. Chapter VII of the new ordinance concerns the protection of human rights of persons who are mentally disordered, and in this manner, it is a great advance. Besides stressing the requirement of informed consent, it grants patients the right to confidentiality, stipulating, “No patient shall be publicised nor his identity disclosed to the public through press or media unless such person chooses to publicise his own condition.” Moreover, suicide, per se, is not to be considered a sign of mental illness, though persons attempting suicide must subsequently be assessed by an approved psychiatrist to ascertain their mental status.

A list of four distinct offences has been created by section 52 (chapter VIII) of the ordinance. These crimes are (1) wilfully making false statements so as to discredit someone as mentally disordered, (2) negligence of a manager of the estate of a person who is mentally disordered or such person's refusal to submit accounts, (3) ill treatment of a patient by the staff of a psychiatric facility, and (4) ill treatment or exploitation, including the traditional practice of induced microcephaly (Box 2), of any person who is mentally ill by members of the public at large. Punishments have been prescribed for these criminal offences, which include fines and imprisonment ranging from six months to five years.

**Box 2. Induced Microcephaly: The Making of “Rat Children”**

According to a legend, infertile women are blessed with children when they pray at the shrine of Shah Dola, a saint buried in Gujrat, Pakistan. But the first-born child in such situations, says the legend, is always microcephalic and must, therefore, be handed over to the custodians of the shrine. The microcephalic children (the “rat children” of Shah Dola) are severely handicapped both mentally and physically, but are considered close to God and, thus, are given charity. It is alleged that this phenomenon is actually the work of criminal gangs, who use iron rings to induce microcephaly in otherwise healthy infants to exploit them as beggars [13].

## Doctors Are Not above the Law

The section of the new ordinance most relevant to the psychiatric profession, perhaps, is section 56 (chapter XI), which deals with specialised psychiatric treatments. It stipulates that all electroconvulsive treatments shall preferably be administered under general anaesthesia and advised by a psychiatrist, not a medical officer or anyone else, and that the reasons for not using other available methods must be recorded.

Also, contrary to popular clinical practice, the ordinance states that “administration of long acting anti-psychotic depot injections shall only be carried out upon the advice of a psychiatrist for a period specified in the prescription and such cases shall be reviewed periodically”. Such stringent measures can help prevent excesses being committed by the profession, though, at times, these restrictions might be found to be overly rigid. Finally, the most stringent controls have been placed on the practice of psychosurgery, obviating any possibility of it being performed, except when found to be absolutely crucial by a comprehensive, stipulated panel of doctors.

Together, the introduction of these measures shows the attention that has been devoted towards streamlining the provision of medical help for individuals who are mentally disordered and towards bridging the gaps in the law. The flip side may be that such detailed legislation might open the floodgates to those who wish to sue psychiatric professionals, though it is likely that such litigation will be rare. There is a lack of professional expertise regarding cases of personal injury caused by medical negligence. Another factor that makes litigation rare is the strong traditional belief in predestination—the belief that ill fate or death is fixed, and thus someone's negligence cannot effect it to a large extent.

## The Road Ahead

The recent changes in the law do signify its dynamic nature. The law's response to changed social and professional attitudes, in the form of the new ordinance, though much belated, is a fitting one. But, as we have indicated in this article, many gaps in the law still remain. Also, steps such as the establishment of the Federal Mental Health Authority can only be judged by performance over the course of the coming years.

We firmly believe that the road ahead in the mental health laws of Pakistan also lies in seeking to comply with international standards. Despite the progress made in comparison with the old law, the current law still falls short of standards in relevant international conventions.

The Universal Declaration of Human Rights [11] and its extension with regard to individuals who are mentally disabled—the Declaration of the Rights of the Mentally Retarded [12]—can be useful guides to action. Briefly put, the latter declaration makes seven guarantees to individuals who are mentally disordered: (1) equal rights to the maximum degree of feasibility; (2) proper education, care, and treatment for self-development; (3) the right to economic security and a decent standard of living; (4) the right to live with one's own family or the closest possible alternative; (5) the right to a qualified guardian, if necessary; (6) protection from exploitation, abuse, and degrading treatment, and restricted civil and criminal liability; and (7) the right that any restriction of rights must be legally monitored, must not be arbitrary, and must be subject to appeal and periodic review.

Although these conventions cannot be incorporated into our domestic law, per se, they do provide standards that we must strive to meet. The recent ordinance has brought us one step closer to such compliance. But many problems still remain, so it has become ever more important that the law be subjected to periodic review by a team of experts who measure its performance with reference to the above-mentioned standards, and suggest the necessary reforms.
